# Genome-Wide Identification of *TCP* Transcription Factors Family in Sweet Potato Reveals Significant Roles of miR319-Targeted TCPs in Leaf Anatomical Morphology

**DOI:** 10.3389/fpls.2021.686698

**Published:** 2021-08-06

**Authors:** Lei Ren, Haixia Wu, Tingting Zhang, Xinyu Ge, Tianlong Wang, Wuyu Zhou, Lei Zhang, Daifu Ma, Aimin Wang

**Affiliations:** ^1^Institute of Integrative Plant Biology, School of Life Sciences, Jiangsu Normal University, Xuzhou, China; ^2^Jiangsu Key Laboratory of Phylogenomics & Comparative Genomics, School of Life Sciences, Jiangsu Normal University, Xuzhou, China; ^3^Xuzhou Institute of Agricultural Sciences in Jiangsu Xuhuai District, Key Laboratory for Biology and Genetic Breeding of Sweetpotato (Xuzhou), Ministry of Agriculture/Jiangsu Xuzhou Sweetpotato Research Center, Xuzhou, China

**Keywords:** *Ipomoea batatas* L., TCP transcription factors, miR319, chloroplast development, expression analysis

## Abstract

Plant-specific TCP transcription factors play vital roles in the controlling of growth, development, and the stress response processes. Extensive researches have been carried out in numerous species, however, there hasn’t been any information available about *TCP* genes in sweet potato (*Ipomoea batatas* L.). In this study, a genome-wide analysis of *TCP* genes was carried out to explore the evolution and function in sweet potato. Altogether, 18 *IbTCPs* were identified and cloned. The expression profiles of the *IbTCPs* differed dramatically in different organs or different stages of leaf development. Furthermore, four CIN-clade *IbTCP* genes contained miR319-binding sites. Blocking *IbmiR319* significantly increased the expression level of *IbTCP11/17* and resulted in a decreased photosynthetic rate due to the change in leaf submicroscopic structure, indicating the significance of IbmiR319-targeted *IbTCPs* in leaf anatomical morphology. A systematic analyzation on the characterization of the *IbTCPs* together with the primary functions in leaf anatomical morphology were conducted to afford a basis for further study of the *IbmiR319*/*IbTCP* module in association with leaf anatomical morphology in sweet potato.

## Introduction

TCP transcription factors (TFs) belong to a small family of plant-specific TFs (Yin et al., 2018) named after its initial members TEOSINTE BRANCHED1 (TB1) in *Zea mays* L. (Doebley et al., 1997), CYCLOIDEA (CYC) in *Antirrhinum majus* L. (Luo et al., 1996), and PROLIFERATING CELL FACTORS 1 and 2 (PCF1 and PCF2) in *Oryza sativa* L. (Kosugi and Ohashi, 1997). TCPs have been ascertained in numerous plant species, such as *Medicago truncatula* Gaertn. (Wang et al., 2018), *Gossypium barbadense* L. (Zheng et al., 2018), *Citrullus lanatus* (Thunb.) Matsum. and Nakai (Shi et al., 2016), *Malus domestica* Borkh. (Xu et al., 2014), *Cucumis sativus* L. (Wei et al., 2014), *Solanum tuberosum* L. (Bao et al., 2019; Wang et al., 2019b), *Solanum lycopersicum* L. (Parapunova et al., 2014), *Populus euphratica* Oliv. (Ma et al., 2016), *Z. mays* (Chai et al., 2017), and *Panicum virgatum* L. (Zheng et al., 2019). Despite the wide-ranging identification of TCPs, just a few of them have been intensively studied. A non-canonical basic-helix-loop-helix (bHLH) motif was at the N-terminal (about 59 amino acids in length) of all the TCP TFs, commonly referred to as the TCP domain. The TCP domain probably associated with the modulation of DNA binding (Kosugi and Ohashi, 2002) as well as protein–protein interactions (Dhaka et al., 2017). Based on the amino acid residue sequences of the TCP domain, TCPs can be categorized into two classes. For class I, it also referred to as the PCF or TCP-P class, while class II referred to the TCP-C class. The consensus DNA-binding site of class I TCPs is GGNCCCAC, however, which of class II is GTGGNCCC (Kosugi and Ohashi, 2002). Furthermore, class II was categorized into two different subclasses: CIN and CYC/TB1. The R domain rich in arginine with unknown function is present in all members of CYC/TB1 and probably mediating protein interactions through a coiled coil (Lupas et al., 1991; Cubas et al., 1999).

Increasing experimental evidence has showed that *TCPs* play a versatile regulatory role at the different stage of plant growth and development, for instance in leaf morphogenesis (Palatnik et al., 2003), trichome formation (Vadde et al., 2018), flower and fruit development (Nag et al., 2009; Koyama et al., 2011), and hormone biosynthesis, as well as in the response to varies stresses (Danisman, 2016; Zhang et al., 2018; Bao et al., 2019; Liu et al., 2019). Twenty-four *AtTCPs* have been identified in *A. thaliana*. AtTCP18, a member of the CYC/TB1 subgroup, regulated two florigen proteins (FT and TSF) to repress the switching from axillary meristems to premature floral (Niwa et al., 2013). AtTCP21 regulated the transcription of *TOC1* and suppresses the expression of *CCA1* to control the circadian clock (Pruneda-Paz et al., 2009; Giraud et al., 2010). Intriguingly, TCP regulated plant growth and development mainly by regulating the biosynthesis of bioactive substances, especially auxin (Challa et al., 2019), ethylene (Liu et al., 2019), gibberellins (Ferrero et al., 2019), brassinosteroids (Guo et al., 2010), jasmonic acid (Danisman et al., 2012), and flavonoids (Viola et al., 2016; Chahel et al., 2019). AtTCP14/15 directly interacted with *GA20ox1*, a key gene in gibberellin biosynthesis, to control the length of petiole and hypocotyl (Ferrero et al., 2019). In addition, AtTCP15 also directly activates the *SAUR63* gene subfamily to participate in gibberellin-dependent stamen filament elongation (Gastaldi et al., 2020). AtTCP15 also serves as a repressor of anthocyanin accumulation by influencing the expression of anthocyanin biosynthesis genes and upstream transcriptional regulators (Viola et al., 2016).

MicroRNAs (miRNAs) are small single-stranded, non-coding RNAs that are typically 20–24 nucleotides (nt) in length. Usually, miRNAs regulate target gene expression through mRNA cleavage at the post-transcriptional level or translational repression (Bartel, 2009; Taylor et al., 2014; Cui et al., 2017). Since most of the target genes of miRNAs are TFs, which can realize the cascade amplification of signals through transcriptional regulation of their target genes, miRNAs have gradually become a potential target gene for crop improvement due to their function in plant growth, development (Axtell et al., 2007), and stress response (Silvestri et al., 2019; Wang et al., 2019a; Kang et al., 2020). In *Arabidopsis*, eight CIN-like *TCP* genes out of 24 *AtTCPs*, including miRJAW–targeted *AtTCP2*, *AtTCP3*, *AtTCP4*, *AtTCP10*, and *AtTCP24*, as well as miRJAW–resistant *AtTCP5*, *AtTCP13*, and *AtTCP17*, were found to chord with different pathways to regulate leaf development (Palatnik et al., 2003; Koyama et al., 2007). The down-regulated expression of *AtTCP2/4* caused by miR319 target-cleavage resulted in serrated leaves (Palatnik et al., 2003). In switchgrass, miR319 negatively regulated *PvTCPs* to promote ethylene accumulation and enhance salt tolerance (Liu et al., 2019). In cotton, high levels of *GhTCP4*, the target of miR319, repressed a homeobox-containing factor, *GhHOX3* transcriptional activity, to promote the secondary cell wall biosynthetic pathway in fiber cells, resulting in shorter fibers and thicker walls (Cao et al., 2020).

Allohexaploid sweet potato, a major global root and tuber crop, is a significant component as to subsistence agriculture because of its capability to guarantee food security and improving nutrition status regionally (Liu, 2014). However, due to the largeness and complexity of the genome, research on the molecular genetics of sweet potato has lagged behind that of other crops, such as rice and potato. Recently, its whole genome was sequenced (Yang et al., 2017). Additionally, its diploid relative wild species *Ipomoea trifida* (Kunth) G. Don and *Ipomoea triloba* L. have also been sequenced (Wu et al., 2018). These genomic sequences can serve as a reference for the hexaploid sweet potato genome. As we still know little about the *TCPs* family in sweet potato, an analysis concerning *TCP* gene family in sweet potato was performed globally in the present study. Eighteen *IbTCP* genes were ascertained and cloned, and phylogenetic relationships, chromosomal locations, and tissue-specific expression were carried out. Furthermore, we found that the transcription patterns of *IbTCPs* discrepant during leaf development. *IbTCP11* and *IbTCP17* were significantly up-regulated in the mature leaves. Blocking IbmiR319 led to an elongated chloroplast and decreased net photosynthetic rate, which further confirmed the critical roles of IbmiR319-targeted *IbTCPs* in leaf anatomical morphology. As a result, our data offers detailed information of *IbTCPs* category and helps elucidate the function of *IbTCPs* in leaf anatomical morphology in sweet potato.

## Materials and Methods

### Plant Materials

The wild-type (WT) sweet potato (*Ipomoea batatas* L.) cultivar “Xushu 22” (Xu22), developed by the Sweet Potato Research Institute of the China Agriculture Academy of Science, was used for *IbTCP* gene cloning. MIM319, overexpressing an artificial IbmiR319 target mimicry to sequester the normal expression of native IbmiR319, was generated using an *Agrobacterium*-mediated embryogenic calli transformation. Untransformed (WT) and transgenic plants transplanted and planted as described before (Yang et al., 2011). The cuttings of 3–4 cm height plants were transplanted grown into the field at the experimental station of Jiangsu Normal University (E 117°17.48′, N 34°16.95′, Jiangsu, China) were used for the assessment of the phenotype and agronomic traits. The plants grown in greenhouses were used for RNA extraction, and those grown in the field were used for morphological observations and photosynthesis parameter measurements.

### Identification and Evolutionary Analysis

The TCP protein sequences of *Arabidopsis* and rice retrieved from the database PlantTFDB^1^ or TAIR^2^ were used to perform protein to protein BLASTP searching with the *e*-value of 10^–5^ in the *I. batatas* genome database^3^ and its two wild ancestors (*I. trifida* and *I. triloba*) genomics database.^4^ All sequences were further validated by the conserved domains database (CDD).^5^ We named *IbTCP1* to *IbTCP18* in the light of their allocations in the genome and relative orders on each chromosome. To study the evolutionary relationships of IbTCPs, Clustal X 2.0 was used to align the entire protein sequences of TCPs from sweet potato, *Arabidopsis*, and rice, which were then used to construct an evolutionary tree in MEGA 6.0 as described before (Tamura et al., 2013).

### Sequence Analysis of IbTCP Genes

Using the ExPASy proteomics server,^6^ the molecular weight (MW) and isoelectric points (pI) of the IbTCPs were predicted.^7^ The conserved motifs of the IbTCPs were ascertained with CDD and the ExPASy proteomics server.

### Subcellular Localization of *IbTCPs*

The coding sequence (CDS) without the termination codon of the *IbTCP11* and *IbTCP17* were amplified through PCR, and then purified and cloned into pCAMBIA2300-35S-eGFP to obtain subcellular localization vectors 35S: IbTCP11-GFP and 35S:GFP-IbTCP17. The *Agrobacterium tumefaciens* EHA105 strain harboring 35S: IbTCP11-GFP or 35S:GFP-IbTCP17 was infected into leaf of *N. benthamiana*. The IbTCP-GFP fusion proteins were detected as described before (Wang et al., 2018). The sequences of primers were also listed in Supplementary Table 1.

### Identification of IbmiR319 and Prediction of IbmiR319 Target Genes

The sequence of IbmiR319 was acquired from our microRNA library of sweet potato based on high-throughput sequencing (Xie et al., 2017; Tang et al., 2020). To forecast the target sites of IbmiR319, the CDS of the *IbTCPs* was analyzed using the psRNATarget online tool.^8^

### Plasmid and Agrobacterium-Mediated Sweet Potato Transformation

The miR319 target mimicry vector p35S-MIM319 was constructed as previously described (Franco-Zorrilla et al., 2007). Genetic transformation of sweet potato was implement as previously described by Yang et al. (2011) to obtained the transgenic plant MIM319.

### Gene Expression Analysis

qRT-PCR was carried out to check the expression profiles of genes in different tissues, including the shoot buds (Sb), young leaf (YL), mature leaf (ML), stem (S), fibrous roots (FR), pencil roots (PR), and developing storage roots (DR), or leaves at the different developmental stages (L1–L10: the 1st through 10th leaves counted from the stem tip). Total RNAs was extracted from the above samples using TRIzol (Invitrogen) as described before (Meng et al., 2018). The inverse transcription and the *qRT-PCR* were performed as described before (Meng et al., 2018). The *IbActin* gene was used as an internal control. Data from three biological samples were collected, and the mean values were normalized to *IbActin*. NRT (no reverse transcription control) and NTC (no template control) were also implemented for each gene analysis. The 2^–ΔΔ*Ct*^ method was used to judge the relative expression level of *IbTCPs* (Livak and Schmittgen, 2001). The sequences for the primers are listed in Supplementary Table 1.

### Leaf Anatomical Morphology

After 2 months of growth, the third fully-expanded leaf counted from the terminal bud was removed and cross sectioned by hand for optical microscopic examinations and measurement of leaf thickness.

For transmission electron microscopy (TEM) observation, the first fully-expanded fresh leaves were resected and cut into small pieces immediately, fixed with electron microscope fixator (Servicebio, G1102, Wuhan Sevier Biotechnology Co. Ltd.). All the procedure were performed according the described online.^9^

### Chlorophyll Content and Photosynthesis Parameter Measurements

The cuttings of WT and MIM319 with four to six expanded leaves transplanted to the field for 4 weeks were used for analysis. The chlorophyll content assay was performed using the third fully-expanded leaves of WT and MIM319 according to the described before (Kuo et al., 2019). The total chlorophyll content was figured out according to the formula in C_*T*_ = 20.29A_645_−8.05A_663_. C_*T*_: total chlorophyll content. A_645_, A_663_: the absorbance of the chlorophyll solution at 645 nm and 663 nm, respectively.

Leaf photosynthesis parameters on the third fully-expanded leaves were scaled using a Li-6400 portable photosynthetic system (Ll-6400, Li-COR) on a sunny day. Ten seedlings per line were scaled.

Chlorophyll fluorescence was measured as described (Kuo et al., 2019). Briefly, after adapting to the dark for 30 min, the *F*_*v*_/*F*_*m*_ values of the third fully-expanded leaves were measured using a Li-6400 system. Ten seedlings per line were measured in total.

## Results

### Identification and Cloning of the *IbTCP* Gene Family in Sweet Potato

**FIGURE 1:**
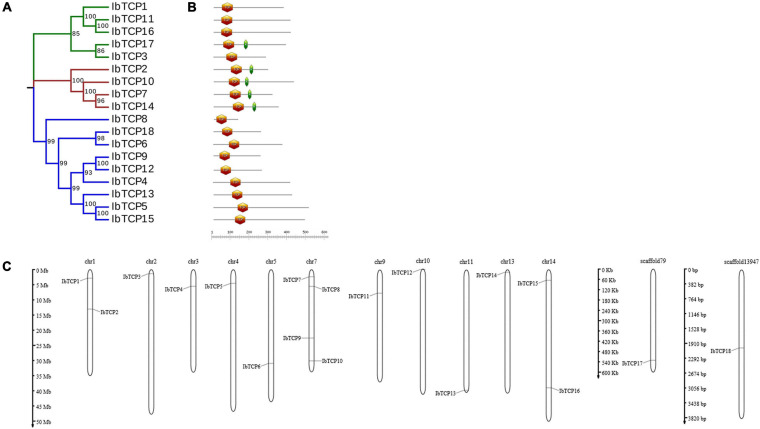
Distribution, phylogenetic tree, and conserved motifs analysis of *IbTCPs* in sweet potato. **(A)** Phylogenetic tree of 18 *IbTCP* genes. The phylogenetic tree was generated using the neighbor-joining method with 1000 bootstrap iterations. Numbers at each node represent the bootstrap values. The blue, green, and red colors indicate the PCF, CIN, and CYC/TB1 clades. **(B)** Conserved motifs analysis. Orange hexagon indicated the TCP domain, and green prism indicated the R domain. The scale refers to the amino acid residues lengths of the TCP proteins. **(C)** Distribution of *IbTCPs* on sweet potato chromosomes or scaffolds. The scale refers to the lengths of the chromosomes or scaffolds in hexaploid sweet potato.

To obtain *TCP* genes in sweet potato, the TCP amino acid sequences of *Arabidopsis* and *O. sativa* were utilized as a reference to BLAST against the hexaploid sweet potato Genome Database, its diploid wild ancestor Sweetpotato Genomics Resource database, and PlantTFDB. After removing the redundant sequences, a total of 18 predicted non-redundant *IbTCP* sequences were identified and further cloned, all of which comprised the conserved TCP domain (Table 1 and Figures 1A,B). Their sequences were listed in Supplementary Table 2. The CDS lengths of the *IbTCPs* varied from 396 bp (*IbTCP8*) to 1527 bp (*IbTCP5*). IbTCP8, with 131 amino acid residues (aa), was regarded as the shortest IbTCP, whereas the largest was IbTCP5 with 508 amino acid residues. The MW was in the range of 14.703–52.774 kDa, and the variation of pI is from 5.53 to 9.83 (Table 1).

**TABLE 1:** Characterization of the *IbTCP* family in sweet potato.

Name	CDS	Length (aa)	Type	MW (kDa)	pI	Chromosomes location	TCP domain location	R domain location
IbTCP1	1110	369	CIN	39.748	6.19	chr1: 2912437-2913561	41-99	/
IbTCP2	873	290	CYC/TB1	32.774	9.00	chr1: 13376720-13377604	94-152	193-210
IbTCP3	846	281	CIN	30.752	9.24	chr2: 1303250-1304098	70-128	/
IbTCP4	1236	411	PCF	43.354	7.37	chr3: 5640446-5641595	94-148	/
IbTCP5	1527	508	PCF	52.774	6.93	chr4: 4619412-4620941	130-184	/
IbTCP6	1110	369	PCF	39.187	5.53	chr5: 31785716-31786825	85-139	/
IbTCP7	942	313	CYC/TB1	35.365	6.81	chr7: 2391142-2392071	87-145	184-201
IbTCP8	396	131	PCF	14.703	9.39	chr7: 5690905-5691300	15-69	/
IbTCP9	753	250	PCF	25.885	9.51	chr7: 23186333-23187085	34-88	/
IbTCP10	1284	427	CYC/TB1	47.716	7.61	chr7: 31005038-31006332	82-140	168-185
IbTCP11	1227	408	CIN	44.293	6.27	chr9: 7933602-7938536	41-99	/
IbTCP12	768	255	PCF	26.970	9.83	chr10: 134409-135176	38-92	/
IbTCP13	1260	419	PCF	43.989	7.45	chr11: 40861501-40903635	100-154	/
IbTCP14	1038	345	CYC/TB1	39.199	9.06	chr13: 786394-787434	104-162	208-225
IbTCP15	1458	485	PCF	50.406	5.86	chr14: 3627481-3629689	115-169	/
IbTCP16	1236	411	CIN	44.629	6.31	chr14: 40034341-40035573	41-99	/
IbTCP17	1155	384	CIN	41.319	6.65	scaffold79: 538826-543936	52-110	162-181
IbTCP18	756	252	PCF	26.823	9.56	scaffold13947: 1620-2378	46-100	/

*aa: Amino acid; pI: the theoretical isoelectric point of proteins; MW: the theoretical molecular weight of proteins.*

To determine gene allocation pattern on chromosome, investigation of the exact genomic positions of them were carried out. These 18 *IbTCPs* were scattered across 11 chromosomes and two scaffolds of sweet potato (Table 1 and Figure 1C). Among them, the *IbTCPs* on chromosomes 2, 10, and 13 were tightly located on the upper end of the arm, while on chromosome 11, they were located tightly on the lower end of the arm. Chromosome 7 contained the highest number of *IbTCPs*, including *IbTCP7*, *IbTCP8*, *IbTCP9*, and *IbTCP10*. Chromosomes 1 and 14 contained two loci each.

### Phylogenetic Analysis and Category of the *IbTCP* Family

**FIGURE 2:**
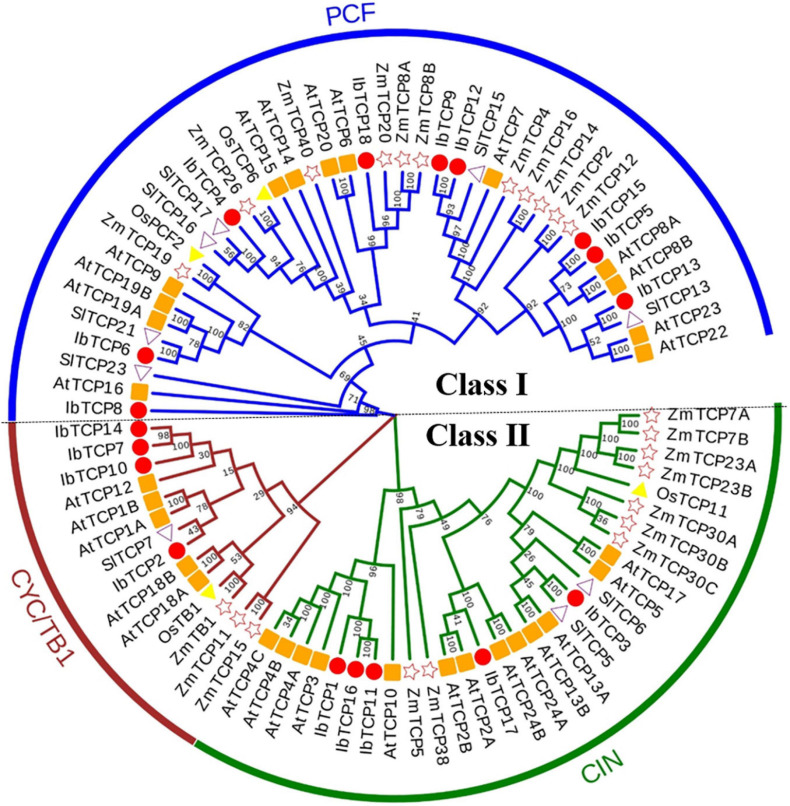
Phylogenetic analysis of TCP TFs from *I. batata* (IbTCP), *Arabidopsis* (AtTCP), rice (OsTCP), maize (ZmTCP), and *S. lycopersicum* (SlTCP). The phylogenetic tree was constructed from 88 full-length protein sequences from *I. batata* (18), *Arabidopsis* (31), rice (7), maize (23), and *S. lycopersicum* (9) using the neighbor-joining method in MEGA 6.0 with 1000 bootstrap replicates.

A phylogenetic tree was structured aimed to investigating about the categorization and evolutionary relationships of sweet potato TCP proteins. A total of 88 TCP full-length amino acid sequences, including 18 IbTCPs, 31 AtTCPs, 23 ZmTCP, seven OsTCPs, and nine SlTCPs, were assembled to structure a phylogenetic tree using the NJ method with 1000 bootstrap replicates (Figure 2 and Supplementary Table 3). In accordance to the categorization in *Arabidopsis*, the IbTCPs may be divided into two TCP classes as well. Nine IbTCPs comprised Class I, while the other nine pertain to Class II, which could be classified into two subclasses: four members of the CYC/TB1 clade and the five members of the CIN clade (Figures 1A, 2). The results suggested that all the TCPs were evolutionarily conserved.

### Conserved Domain Analysis and Recognition Sequence of miR319

**FIGURE 3:**
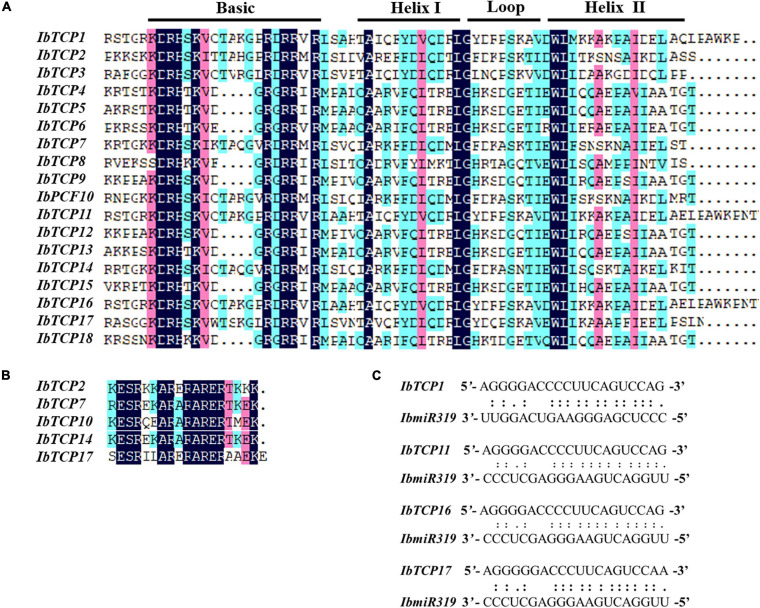
Multiple sequence alignment of IbTCP proteins and the IbmiR319-targeted IbTCPs. **(A)** Alignment of the TCP domain of the 18 TCP proteins in sweet potato. Shaded in different colors indict the conserved amino acids. Conserved domains, including Basic, Helix I, Loop, and Helix II, are shown at the top. **(B)** Alignment of the R-domain of the class II members. Shaded in different colors indict the conserved amino acids. **(C)** Complementarities of IbmiR319 and its target *IbTCP1*, *IbTCP11*, *IbTCP16*, and *IbTCP17* mRNAs.

To further comprehend the evolutionary relationships of IbTCPs in sweet potato, the domains of the IbTCPs were confirmed using the ScanProsite tool.^10^ As expected, all 18 IbTCPs displayed a highly conserved TCP domain that incorporated a bHLH-type motif located near the N-terminal (Figures 1B, 3A). There’s a large difference in the components of the loop and helixes I and II between the class I and II. By analyzing the phylogenetic tree and aligning the TCP domains, the IbTCP proteins are anticipated to be categorized into two classes (Figure 1A), as has been indicated for all species analyzed up to now. The conserved R domain was only found in IbTCP2, IbTCP7, IbTCP10, IbTCP14, and IbTCP17, all of which are members of Class II (Figures 1B, 3B).

MicroRNA319 (miR319) pertains to one of the most ancient and conserved miRNA families. Previous studies have verified that miR319, targeting TF *TCP* genes, plays important roles in plant growth, morphogenesis, and reproduction (Palatnik et al., 2003; Sun et al., 2017; Cao et al., 2020). The IbmiR319 target sites among the 18 *IbTCPs* were analyzed using psRNATarget online. The putative recognition sites of IbmiR319 were found in *IbTCP1*, *IbTCP11*, *IbTCP16*, and *IbTCP17* (Figure 3C). These four *IbTCP* genes all belonged to class II, which corroborates a previous study in *Arabidopsis.* Although mismatches existed at the 3′ end of the IbmiR319 and 5′ end of the targeted *IbTCP*, kernel sequences (3′-GGGAAGUCAGGU-5′) were conserved (Figure 3C). These data illustrate that miR319 maintained homologous target interactions in time of the evolution and diversification of plants.

### Expression Profiles of *IbTCP* Genes in Different Tissues

**FIGURE 4:**
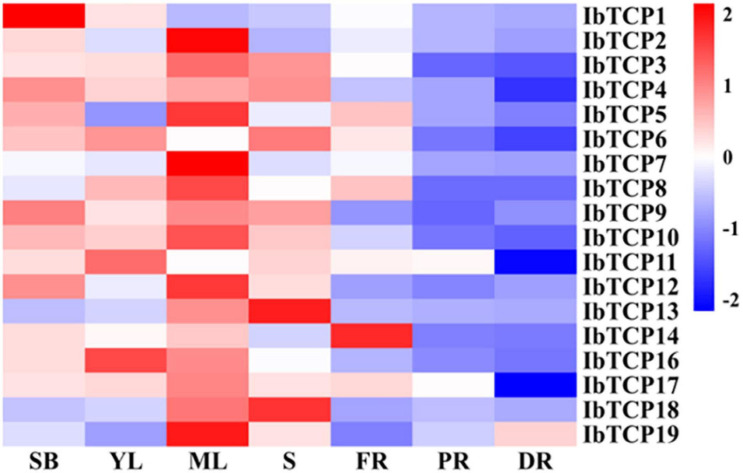
Heatmap representation of the gene expression of 18 *IbTCPs* in various tissues. The tissues used for transcription pattern analysis are listed at the bottom, shoot buds (SB), young leaves (YL), mature leaves (ML), stems (S), fibrous roots (FR), pencil roots (PR), and developing roots (DR). The genes are listed on the right of the expression bars.

To obtain credible information of the growth and developmental functions of *IbTCP* in sweet potato, their organic-specific expression patterns, containing the shoot buds, young leaf, mature leaf, stem, fibrous roots, pencil roots, and developing storage roots, were analyzed by qRT-PCR. As indicated in Figure 4, even though all of *IbTCPs* were expressed in all seven tissues tested, there’s considerable variation in transcription levels of different genes among different tissues. Generally speaking, the expression levels of *IbTCPs* were relatively low in the belowground organs, whereas constitutively high expression in the aboveground organs examined, especially ten IbTCPs (*IbTCP2*, *IbTCP3*, *IbTCP5*, *IbTCP7, IbTCP8, IbTCP9*, *IbTCP10*, *IbTCP12, IbTCP17*, and *IbTCP19*) showed highly expression levels in the mature leaves. This finding implies that these *IbTCP* genes probably perform different functions during growth and development. Interestingly, the relatively higher expression of all the IbmiR319-targeted CIN subclass *IbTCPs* (*IbTCP1*, *IbTCP11*, *IbTCP16*, and *IbTCP17*) were detected in the shoot bud, young leaf, and mature leaf, while expression levels of the other *IbTCPs* were high mainly in the mature leaf, suggesting that they may play similar or different roles in leaf development.

### IbmiR319-Targeted *IbTCPs* Play Vital Roles in Leaf Anatomical Morphology

**FIGURE 5:**
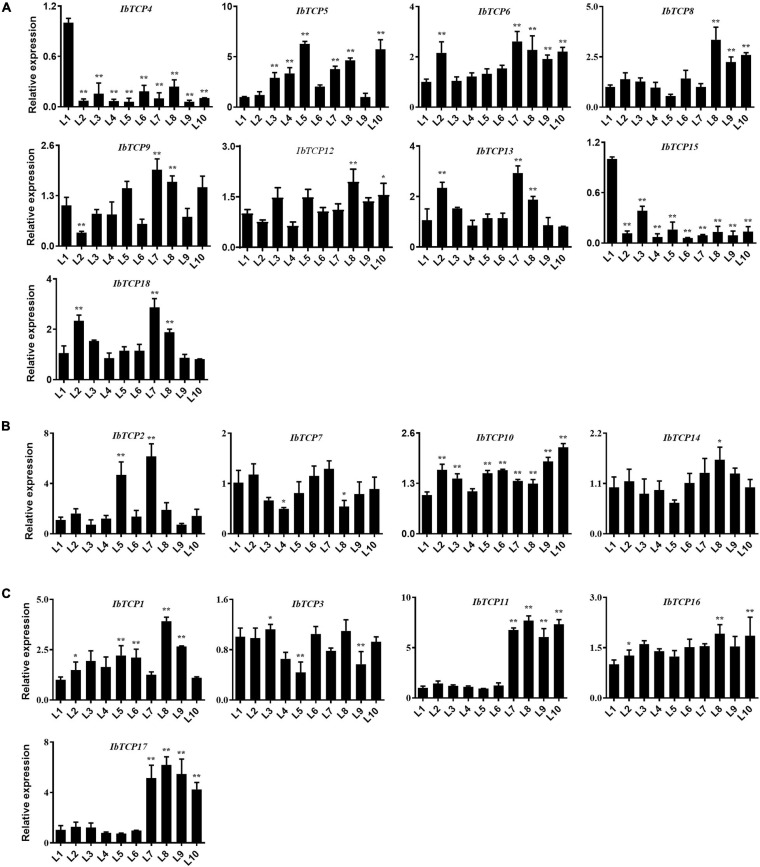
Expression pattern analysis of *IbTCPs* in varying leaf developmental periods. The qRT-PCR transcript analysis of *IbTCPs* of Class I **(A)**, Class II CIN clade **(B)**, and CYC/TB1 clade **(C)** in the 1st, 2nd, 3rd, to 10th leaf (L1–L10). The expression profiles were normalized using *IbActin*. Values are the mean and SD of three replicates. The asterisks indicate statistical significance to L1 (Student’s *t*-test; *P < 0.05, **P < 0.01).

Leaves, as the major photosynthetic organs of plants, take up a crucial position in plant growth and development. Leaves are also important for tuber crops, since the photosynthetic rate directly affects dry matter accumulation and storage root swelling (Sawada et al., 1999). To understand the function of *IbTCPs* in leaf development, their transcription levels of 18 *IbTCPs* at the different developmental stages of the leaves were investigated (Figure 5). The expression levels of most *IbTCPs* exhibited no signification changes at different developmental stages (Figures 5A,B). Yet, the transcription levels of IbmiR319-targeted *IbTCPs*, especially *IbTCP11* and *IbTCP17*, presented a significant increase (Figure 5C). These data indicate that the IbmiR319-IbTCPs module is likely to associate with leaf development.

**FIGURE 6:**
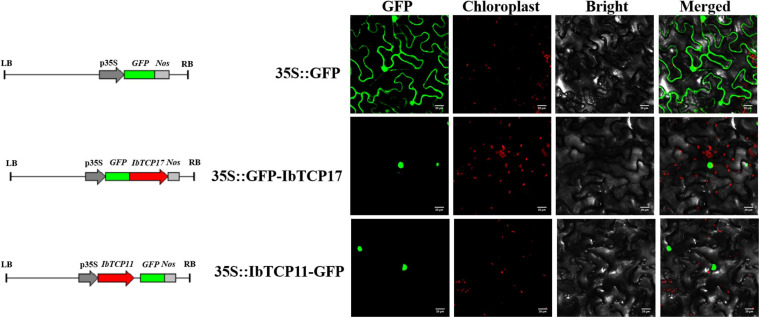
Subcellular localization of two GFP-fused sweet potato TCP proteins. The two IbTCP-GFP fusion proteins (IbTCP11-GFP and IbTCP17-GFP) were transiently expressed in tobacco leaves and observed by confocal microscopy 72 h later. GFP driven by the 35S promoter was used as a control. The left, middle-left, middle-right, and right panels stand for photoes taken in dark, chloroplast, bright, and merged views, respectively. Bars are 20 μm.

For a more thorough evaluation of the duty of the IbmiR319-IbTCPs module, the subcellular localization of the IbmiR319-targeted *IbTCPs* was performed. *IbTCP11* and *IbTCP17* were fused with green fluorescence protein and infiltrated into *N. tabacum* epidermal cells. According to the distribution of green fluorescence signal, these two IbmiR319-target *IbTCPs* were specifically lay within the nuclei, implying that they are functional TFs (Figure 6). The IbmiR319 target mimicry vector p35S-MIM319 was then constructed and introduced into WT, and the stable transgenic line MIM319, which blocked IbmiR319, was generated (Supplementary Figure 1A). The expression levels of *IbTCP11* and *IbTCP17* were dramatically up-regulated in MIM319 compared with WT (Supplementary Figure 1B), indicating that these two *IbTCPs* were the targets of IbmiR319.

**FIGURE 7:**
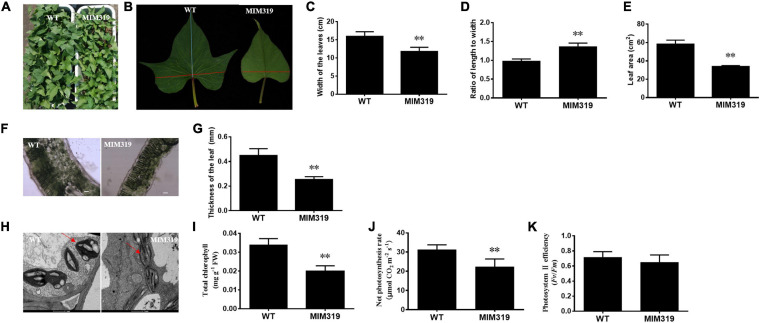
The phenotypes and anatomical morphology of MIM319 and WT sweet potato leaf. **(A)** Phenotype of the whole plant of WT and MIM319. **(B)** Phenotype of the third fully-expanded leaf of WT and MIM319. **(C–E)** The width, ratio of length to width, and area of the third fully-expanded leaf of WT and MIM319. **(F)** The microstructure of the hand-sliced third-fully expanded leaves of WT and MIM319 observed using an optical microscope. Bars are 50 μm. **(G)** The thickness of the third fully-expanded leaf of WT and MIM319. **(H)** The TEM image of the third fully-expanded leaf of WT and MIM319. The red arrows indicate the chloroplast. Bars indicate 1 μm in length. **(I–K)** The total chlorophyll, net photosynthesis rate, and photosystem II efficiency of WT and MIM319. The error bars and asterisks indicate the SD and statistical significance of 10 biological replicates (Student’s *t*-test; ^∗∗^*P* < 0.01), respectively.

The up-regulation of *IbTCP11* and *IbTCP17* caused by blocking IbmiR319 in sweet potato led to dramatic changes in leaf size and leaf shape (Figures 7A,B). Quantitative analysis revealed that the width of the third fully-expanded leaves of MIM319 transgenic sweet potato ranged from 14.7 to 18.0 cm, which was dramatically less than that of WT (Figure 7C), and the leaf area ranged from 55.50 to 63.25 cm^2^, which was also significantly less than that of WT (Figure 7E). Conversely, there was no obvious difference in the length of the fully-expanded leaves between MIM319 and WT (Supplementary Figure 2). As a result, the ratio of the length to width of MIM319 was greater than that of WT (Figure 7D). Taken together, the data indicated that IbmiR319-targeted *IbTCPs* may have conserved functions during leaf development, similar to their functions in *Arabidopsis* and rice (Palatnik et al., 2003; Nag et al., 2009; Yang et al., 2013).

To elucidate the cellular basis of the morphological changes, microstructure and ultrastructure observations of the leaves were carried out by electron microscopy using hand-sliced paraffin sections. Microscopic analysis of the leaf blade cross-sections revealed fewer mesophyll cells (including the palisade tissue and spongy tissue) and thinner layers of mesophyll cells in MIM319 than in WT (Figure 7F). The thickness of MIM319 leaves (0.264 mm on average) was significantly thinner than that of WT (0.464 mm on average) (Figure 7G). The chloroplasts were flatter, more elongated, and contained less starch grains compared with WT (Figure 7H). In combination, the data indicated that IbmiR319-target *IbTCPs* also play vital roles in leaf anatomical morphology.

Leaf size and structure are important since they impact photosynthetic efficiency (Marcotrigiano, 2010). Thinner leaves with less chlorophyll content are considered to have a detriment for the efficient usage of light energy, and lead to less photosynthetic efficiency (Higuchi et al., 1999). To address whether the changes in leaf anatomical morphology of MIM319 affect photosynthesis, some photosynthetic parameters were quantitatively measured. The total chlorophyll content of the MIM319 transgenic plants was 0.0199 mg g^–1^ FW on average, while that of WT was 0.0331 mg g^–1^ FW. The total chlorophyll contents of MIM319 were dramatically lower than that in WT (Figure 7I). Furthermore, the photosynthetic rate of the MIM319 transgenic sweet potato was 22.33 μmol CO_2_ m^–2^ s^–1^ on average, which was dramatically lower than 31.24 μmol CO_2_ m^–2^ s^–1^ in the WT (Figure 7J). Meanwhile, the efficiency of PSII in MIM319 transgenic sweet potato leaves was also examined based on the *F*_*v*_/*F*_*m*_ values. However, there were no significant differences between MIM319 and WT (Figure 7K). Taken together, these data suggested that IbmiR319-targeted *IbTCPs* may play key roles in the anatomical morphology of the leaves to affect photosynthesis.

## Discussion

As plant-specific TFs, TCPs have major functions during growth and development, such as leaf (including single leaf and compound leaf) development, shoot development, flower development (Ori et al., 2007; Broholm et al., 2008; Koyama et al., 2011; Bai et al., 2012), and root development (Hao et al., 2012), and are also involved in phytohormone biosynthesis (Danisman et al., 2012), lignin biosynthesis (Sun et al., 2017; Chahel et al., 2019) and the circadian clock (Giraud et al., 2010; Zhang et al., 2018). Nevertheless, there’s scarcely any precise exhaustive information of the TCP TF family in sweet potato which is the seventh largest food crop in terms of production globally and a vital tuber crop. In this study, 18 *IbTCPs* were identified and cloned in sweet potato and were discovered to be distributed on 11 chromosomes and two scaffolds with different densities (Figure 1C). Phylogenetic analysis revealed that 18 IbTCPs clustered into two classes according to their evolutionary relationships (Figures 1A, 2). These findings are consistent with previous classifications of TCPs from *Arabidopsis*, *Z. mays*, and *S. lycopersicum* (Kim et al., 2014; Parapunova et al., 2014; Chai et al., 2017). Conserved motif analysis showed that TCP domain with a bHLH-type motif existed in both class I and class II of IbTCPs, while the R domain only existed in class II (Table 1 and Figures 1B, 3A,B). Collectively, this evidence supports the classification and conservation of the sweet potato TCP TF family.

It is well-known that class II TCPs precisely regulate the transition from division to expansion (Tsukaya, 2010; Qi et al., 2017). For the leaf lamina, the timing of the transition is a significant determinant of the final size, shape, flatness, and complexity. Class II CYC/TB1 clade TCPs basically participate in the developmental regulation of axillary meristems, which results in the growth of either lateral branches or flowers. This clade included AtTCP2/3/4/5/10 and IbTCP11/17 (Figure 2). We therefore examined the expression patterns of *IbTCPs* to investigate whether they have essential functions in the growth and development of these organs or tissues. The expression in different tissues and different organs varied widely among *IbTCP* genes and different development stages of the leaf for individual *TCP* genes (Figures 4, 5). This states the functional divergence of *IbTCPs* at different stages of developmental processes, especially leaf development in sweet potato. In our study, all 18 *IbTCPs* showed relatively weak expression in the belowground organs, but high expression in the aboveground organs, with even *IbTCP11* and *IbTCP17* showing higher expression in the mature leaves (Figure 5C). *AtTCP3* and *AtTCP4*, orthologs of *IbTCP11*, act as decisive modulators of *de novo* shoot organogenesis (Woorim et al., 2020). A previous study demonstrated that the overexpression of *AtTCP4* led to in miniature cotyledons and shoot apical-meristem termination (Efroni et al., 2008), and the ectopic expression of *AtTCP3* caused the failure of shoot-meristem formation (Koyama et al., 2007). Double, triple, or even quadruple knockouts for the miR319-regulated *TCP* genes, *AtTCP2*, *AtTCP3*, *AtTCP4*, and *AtTCP10*, demonstrate leaf crinkling to varying degrees, yet single knockouts present only slight effects on leaf morphology (Schommer et al., 2008; Bresso et al., 2018). *AtTCP5* represses the initiation and outgrowth of leaf serrations by directly promoting the expression of *KNAT3* and indirectly activating the transcription of SAW1 (Yu et al., 2020). Together, these data reveal the tissue expression diversity of *TCPs* in various plants. Compared with other plants, the great number of highly-expressed *IbTCPs* in the aboveground organs indicates their important function in sweet potato development, especially leaf development.

Previous study recorded that TCPs are fundamental regulators of plant growth and development, especially leaf development and senescence (Schippers, 2015). The leaf is the pivotal organ of photosynthesis, and its size and anatomical morphology directly affect photosynthetic efficiency (Marcotrigiano, 2010; Pan et al., 2018). Many genes and pathways can regulate leaf morphogenesis so as to generate organs of a wide variety of sizes and shapes (Rodriguez et al., 2014). Meanwhile, more and more experimental data show that miRNAs regulate the TFs which involved in plant development. The MiR164 family includes post-transcriptionally regulated *CUC1*, *CUC2*, and other *NAC* TFs. The overexpression of the miR164 family in *Arabidopsis* compromised organ separation, causing fusion between cotyledons and leaves, and also between the inflorescence stem and cauline leaves (Laufs et al., 2004; Mallory et al., 2004; Nikovics et al., 2006). MiR165/166 precisely regulated the expression pattern of polarity genes during leaf development in maize (Juarez et al., 2004). Increased miR408 expression leads to enhanced photosynthesis by elevating the abundance of plastocyanin to improve irradiation utilization efficiency in *Arabidopsis*, tobacco, and rice (Pan et al., 2018). This study indicates that blocking miR319 in sweet potato increases *TCP* gene expression to limit photosynthetic performance through thinner and flatter chloroplasts, a lower chlorophyll content, and the development of thinner and smaller leaves.

Our findings provide evidence for the connection between structure and function in the *IbTCPs* as well as lay a basis for further illustrating the functions of *IbTCPs* and their relationship with miR319 in sweet potato.

## Data Availability Statement

The original contributions presented in the study are included in the article/Supplementary Materials, further inquiries can be directed to the corresponding authors.

## Author Contributions

LR, LZ, DM, and AW designed the research. LR, HW, and TZ implemented most of the research and analysis. XG, WZ, and TW implemented *IbTCPs* identification, cloning, and bioinformatics analysis. LZ contributed analytical tools. LR, LZ, and AW wrote the manuscript. LZ and DM modified the manuscript. All authors contributed to the article and approved the submitted version.

## Conflict of Interest

The authors declare that the research was conducted in the absence of any commercial or financial relationships that could be construed as a potential conflict of interest.

## Publisher’s Note

All claims expressed in this article are solely those of the authors and do not necessarily represent those of their affiliated organizations, or those of the publisher, the editors and the reviewers. Any product that may be evaluated in this article, or claim that may be made by its manufacturer, is not guaranteed or endorsed by the publisher.

## Footnotes

1
http://planttfdb.gao-lab.org/

2
https://www.arabidopsis.org/

3
http://public-genomes-ngs.molgen.mpg.de/SweetPotato/

4
http://sweetpotato.plantbiology.msu.edu/

5
https://www.ncbi.nlm.nih.gov/Structure/cdd/wrpsb.cgi

6
https://prosite.expasy.org/scanprosite/

7
https://web.expasy.org/compute_pi/

8
http://plantgrn.noble.org/psRNATarget/home

9
https://www.servicebio.cn/data-detail?id=4305&code=DJSYBG

10
https://prosite.expasy.org/prosite.html

## Supplementary Material

Supplementary Figure 1 The expression of IbmiR319 **(A)** and its target genes IbTCP11, 17 **(B)** in MIM319.

Supplementary Figure 2 The length of leaf in MIM319.
