# Supplementary figures and images for: Genome-Wide Identification of TCP Transcription Factors Family in Sweet Potato Reveals Significant Roles of miR319-Targeted TCPs in Leaf Anatomical Morphology
Source: Front Plant Sci. 2021 Aug 6;12:686698. doi: 10.3389/fpls.2021.686698 (PMC8379018; doi:10.3389/fpls.2021.686698)

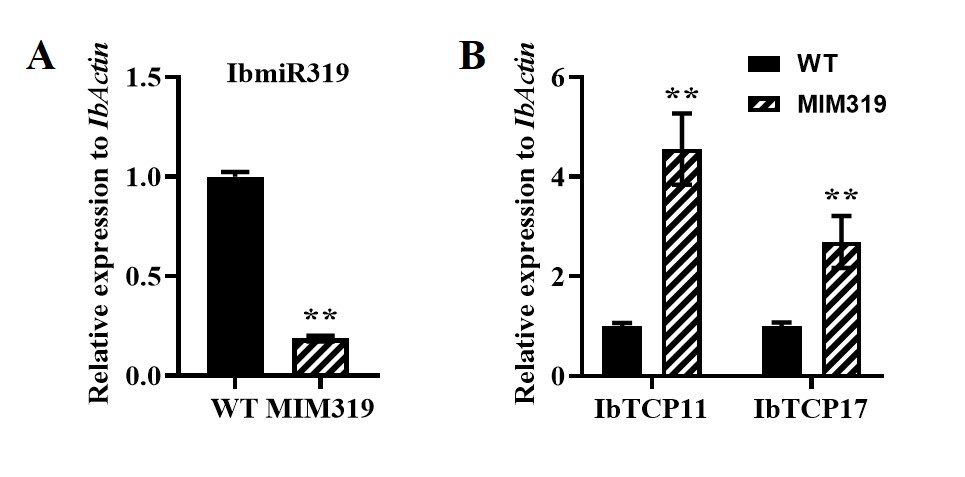

Supplement: Supplementary Figure 1 — The expression of IbmiR319 (A) and its target genes IbTCP11, 17 (B) in MIM319. [file Data_Sheet_1.ZIP › Supplementary File(s)/Fig S1.jpg]

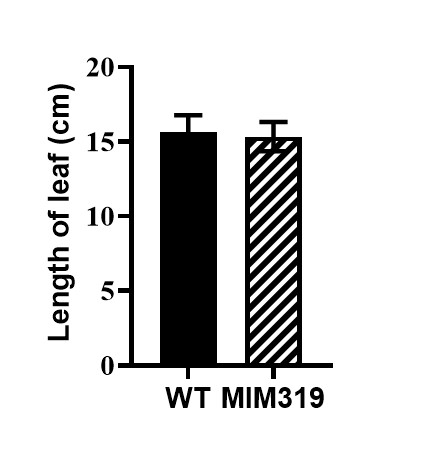

Supplement: Supplementary Figure 1 — The expression of IbmiR319 (A) and its target genes IbTCP11, 17 (B) in MIM319. [file Data_Sheet_1.ZIP › Supplementary File(s)/Fig S2.jpg]
